# Learning What to Want: Context-Sensitive Preference Learning

**DOI:** 10.1371/journal.pone.0141129

**Published:** 2015-10-23

**Authors:** Nisheeth Srivastava, Paul Schrater

**Affiliations:** 1 Dept of Psychology, UC San Diego, La Jolla, CA, United States of America; 2 Depts of Psychology & Computer Science, University of Minnesota, Minneapolis, MN, United States of America; Middlesex University London, UNITED KINGDOM

## Abstract

We have developed a method for learning relative preferences from histories of choices made, without requiring an intermediate utility computation. Our method infers preferences that are rational in a psychological sense, where agent choices result from Bayesian inference of what to do from observable inputs. We further characterize conditions on choice histories wherein it is appropriate for modelers to describe relative preferences using ordinal utilities, and illustrate the importance of the influence of choice history by explaining all major categories of context effects using them. Our proposal clarifies the relationship between economic and psychological definitions of rationality and rationalizes several behaviors heretofore judged irrational by behavioral economists.

## Introduction

Where both psychology and economics unite in principle is their interest in the preferences of humans. While it is generally agreed that people form preferences through experience [1], in the absence of both theory and data, it has historically proved most useful to treat preferences as static and exogenous model inputs.

But by doing so, we lose information about individual differences in agent decisions, many of which are systematic and consequential. For example, rising prices might price poor households entirely out of the market for a good, while incentivizing rich households to pull forward demand for the good to hedge against further price rises. Thus, inflation can both suppress and promote current demand, depending on household’s current wealth levels. Similarly, some people will save large unexpected windfalls (such as direct financial transfers), reducing the money supply, while others will spend them immediately, boosting demand.

Modelers currently account for this diversity in human behavior by making parametric assumptions about the range of risk attitudes present in the population, then adjusting the representative agent predictions accordingly to account for this artificially induced behavior heterogeneity. Thus, in the absence of formal models of how peoples’ preferences originate, economic models are forced to rely upon regressions on conveniently available variables to exogenously induce variety into their behavior, reflecting real world behavior diversity.

Because the efficacy of costly economic interventions depends crucially on benefits predicted by such economic models, the absence of any useful theory of real human behavior to guide them is a glaring deficiency. While early economists were justified in being skeptical of contributions from the inchoate field of psychology, neoclassical economics’ continued indifference to psychological models of behavior has been questioned in light of contributions that behavioral economists have made to the economic literature. As Camerer [2] points out, the“only active resistance to behavioral economics is based on the pessimistic fear that the psychological evidence is too fragmented to suggest coherent **formal** alternatives to rationality.” (emphasis added).

We suggest that the proliferation of ever more fine-grained behavioral data online permits the introduction of a formal theory of preference dynamics, that can usefully guide economic preference modeling. The desiderata for such a theory are formidable: it must draw upon empirical insights about preference dynamics obtained by psychologists, it must be formally tractable so it fits existing microeconomic theory well, and it must make empirical contact with the type of data that is econometrically available.

In this paper, we show that satisfying these requirements is possible and construct an inductive account of preference formation that yields, as a special case, economically rational preferences. Responding to economists’ pessimism regarding the profusion of latent variables that often plague psychological theories, our theory operates with observable inputs (subject choices among options *currently available* to them) and yields measurable outputs (choice probabilities).

The principal contribution of this work is the development of a formal model that **infers** the relative desirability of options from a prior history of choices using Bayesian learning. Critically, this theory permits observers to learn from past choices without assuming that they are either assigning hedonic ‘utility’ to options, or even comparing them with other options available. By designing such a theory, we provide both a principled alternative to assuming exogenous preferences and kill two related inter-disciplinary birds with one stone.

One, we present a novel solution to the psychologists’ question: where do preferences come from? There is an increasing recognition in the psychology and neuroscientific communities [3] that assuming psychophysical access to ‘value’ is problematic—both behavioral and neurophysiological studies have yielded results that contradict such assumptions [4]. By developing a theory of preference formation without recourse to value, we solve this problem at a fundamental level.

Two, we characterize the set of psychological experiences that existing economic definitions of preferences can model, and show how a variety of effects that behavioral economists recognize as ‘biases’ arise simply from applying economic preference models to psychological experiences that live outside this set. Our more general model of preference behavior rationalizes such behaviors, grounding them in differences of choice history that are well-documented in behavioral economics. By clarifying the ontological commitments of classical economics to human behavior within a formal model, our theory thus also shows a clear path to bridging the divide between economic theory and experimental findings.

A brief note about terminology: throughout this paper, we use the term **desirability** to denote a learned sense of what to do. **Relative desirability** can be interpreted as the probability of choosing one from amongst a set of options. **Relative preferences** correspond to relative desirability measurements for an entire choice set that are complete and transitive in the traditional economic sense.

## Preference learning from choice history

*Whatever has value in our world now does not have value in itself, according to its nature—nature is always value-less, but has been given value at some time, as a present—and it was we who gave and bestowed it*.

Friedrich Nietzsche, *The Gay Science*

The idea that preferences are learned from experience has been previously suggested within economics, particularly at the interface between microeconomics and consumer marketing research [1, 5]. Psychologists too have long understood the mechanisms of habituation, conditioning and reinforcement [6, 7], whereby animals’, and hence humans’, preferences are shaped by the consequences of their prior experience.

But it is not enough for us to be able to say that someone is attracted to option *x* more than before because of a signal *r*, i.e. r ⇒ *x*_now_ ≻ *x*_before_, which is what current accounts of preference learning like reinforcement learning can tell us [8]. It has proved difficult to develop models of behavior that can generate preferences without imbuing individual options with some numerical notion of value *r*, i.e. cause = *r*(*x*) > *r*(*y*), *r* : *x* → ℝ_+_. Thus, existing formal models of preference formation end up assuming what they are supposed to generate—that value ‘signals’ associated with an option pre-exist in the environment, and that the goal of a theory of preference formation is to specify how to efficiently separate out these signals from ambient noise introduced by probability, as proposed, for instance, in the signal detection account of utility maximization [9].

If how much something is valued is already a signal though, then a person’s experience cannot have a role in shaping it, which contradicts the primary role that psychological theories assign to experience. So we arrive at an impasse. Existing models of preference formation can either incorporate the role of experience in shaping the desirability of one object, or ignore the role of experience and estimate some fixed preference relation across multiple objects. It is this theoretical impasse that we break in this paper—we develop a formal model that allows preferences to change over time, as psychological theories of conditioning show they must, but also permits inter-option preference comparisons.

The intuitive crux of our new approach is that we no longer assume that preferencs indicate option *superiority*, but simply *acceptability* within the specific context of the choice. By this premise, someone can choose an option out of a set without considering it *superior* to the other options [10]. They may choose it because someone else asked them to, because it was closest at hand, farthest to hand, in the middle of the display etc. All that matters for our account is that they chose it. Thus, our definition of outcome *acceptability* decouples choice behavior from any underlying hedonic maximization principle.

The psychological provenance of *acceptability* need not be restricted simply to the observer’s experience. While even an infant knows a noxious stimulus, say a vaccine injection, when it is encountered, the typical adult expresses preferences and opinions for reified objects far removed from realms where biological signals suffice to signal *acceptability* or vice versa. *Acceptability* for such objects is learned via social mechanisms, beginning with parental guidance and expanding via associative connections to encompass the rituals of daily existence. Thus, in general, our model considers social signaling mechanisms as legitimate sources of information for the *acceptability* signal. However, in the interest of making testable predictions, this paper focuses on the role of individually decipherable choice histories in shaping preferences, ignoring the role of social learning.

### From memories of acceptability to relative preferences

We devote this section to formalizing the key epistemic assumptions underlying our theory. The most distinctive feature of our approach, as we describe above, is a decoupling from the option comparison requirements that form the axiomatic foundations of most preference models. In particular, we only assume people can identify (possibly non-uniquely) items that are *acceptable*. However, acceptability is not a universal property associated with the item, choices acceptable in one context may be unacceptable in another.

Decision contexts are a possibly complex set of additional domain restrictions and requirements that we index by a discrete context variable. Contexts serve the same purpose as cases in case-based decision theory [11], they permit differential accumulation of decision evidence. Each past decision has an associated memory structure, indexed by a context variable *c* ∈ C, where C is a finite set of *contexts*. For each context, the associated memory provides an acceptibility function *R*_*c*_(*x*), and a rate function *A*_*c*_(*x*) which defines the option availability. These are formalized below:

**A1 Acceptability**: For every remembered instance of decisions in a context, decision makers have an indicator function *R*_*c*_(*x*) = **1**_*R*_*c*__(*x*) that is 1 for acceptable options and 0 for unacceptable.

For multiple experiences with decisions in the same context, the acceptability function *R*_*c*_(*x*) will have a stochastic counterpart, *p*(*r*∣*x*, *c*), reflecting the balance of historical evidence in favor of the acceptability of item *x* in decision context *c*, and which plays a central role in our theory. Anticipating its centrality, we call it the *desirability* probability.

**A2 Availability**: For every context, the memory of option availability is represented by an indicator function **1**_*A*_*c*__(*x*) that is 1 for options remembered and 0 for those not.

The intuition for this assumption is that options will be available in a decision context via a stochastic process that determines the number and kinds of options available. These rates depend on a complex set of factors that the decision-maker may or may not be able to model. The memory of a particular decision instance will have an observed set of options *o*, availability evidence accumulated across multiple exposures to decisions in the same context will generate a stochastic counterpart *p*(*x*∣*c*) for the rate function *A*_*c*_(*x*). We call this the *observation* probability.

For new decision instances, we construct a predicted acceptability function that combines across memories of item acceptability in different contexts. To make this prediction, we assume that the probability of recall is a monotonic function of the similarity between a new and previous decision instances. In general this similarity may be a function of item or context attributes. Here we assume a minimal similarity function based on the overlap between item sets across contexts i.e. *menu overlap*. The item-based context similarity function is a map *w* = *sim*(*x*_*c*_1__, *x*_*c*_2__) between sets of items *x*_*c*_1__ and *x*_*c*_2__ ∈ X from contexts *c*_1_ and *c*_2_ to *w* ∈ *W*, with the following properties. The similarity between disjoint item sets is zero, *sim*(**a**, **b**) = 0, if **a** ∩ **b** = ∅. Similarity is reduced as overlap decreases *sim*(**a**, **a** ∩ **b**) ≤ *sim*(**a**, **a**). With these conditions, our memory similarity function will assign progressively lower probabilities as the overlap between sets reduces.

**A3 Memory Similarity**: For options *o* in a new decision instance, the probability a stored context will be recalled is a monotonic function of the number of items overlapping between *o* and *x*_*c*_.

This assumption implies a probability function *p*(*c*∣*o*) that represents the user’s inference about the relevance of experienced contexts to the current decision instance, and which we call a *context* probability.

**Fig 1:**
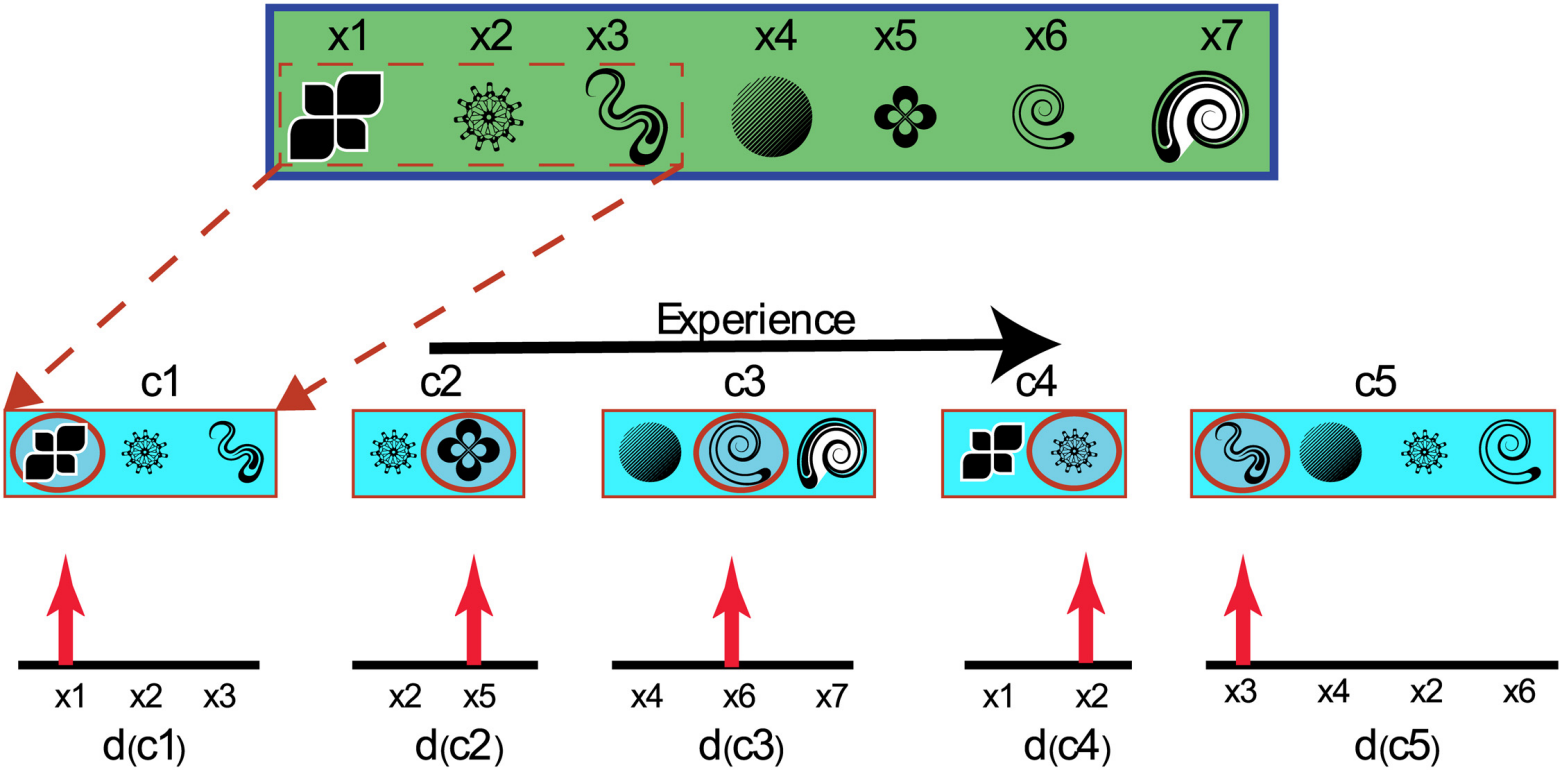
Illustrating the flow of information in typical choices. Options **(x)** co-occur in subsets, within particular contexts **(c)** of observation. Thus option sets themselves are indicative of context. Information about the acceptability of options is confined to desirability functions **(d)** that point to acceptable options in a given context.

Together, these three assumptions imbue a decision maker with the probabilistic knowledge needed to compute the probability options in *o* are acceptable, which we term *desirability*. A schematic illustrating the relationships between these knowledge representations is shown in Fig 1.

### Inferring desirability across contexts

Having outlined the conceptual building blocks of our approach, we now describe the probabilistic dynamics that permit observers to update their world knowledge through the sequential experience of choices, and construct relative preferences for new observations based by drawing upon their memory of previous choices. Our theory focuses on partitioning evidence that a particular choice is desirable into context-specific evidence pools, a strategy that has previously been used by [11] in developing case-based decision theory (CBDT). Our theory can be viewed as an inductive generalization of CBDT, and so is perhaps best explained as such.

In CBDT, subjects maintaing memories of previous choice experiences as a tuple 〈*z*, *a*, *r*〉 denoting respectively problem, action and result. Since we focus on discrete-choice settings, problem description is equivalent to contexts, and actions are equivalent to choosing options, so the CBDT memory tuple can be harmonized in our notation as 〈*c*, *x*, *r*〉.

The CBDT utility calculation assigns different utility values *u*(*r*) to the results in different cases, then determines a holistic utility function for actions *x* by summing across these case-specific utilities weighted by the similarity of their related contexts to the current observation *o*, *U*(*x*) = ∑_〈*c*, *x*, *r*〉 ∈ *M*_ *s*(*c*, *o*)*u*(*r*), where *s* is a context-similarity function.

**Fig 2:**
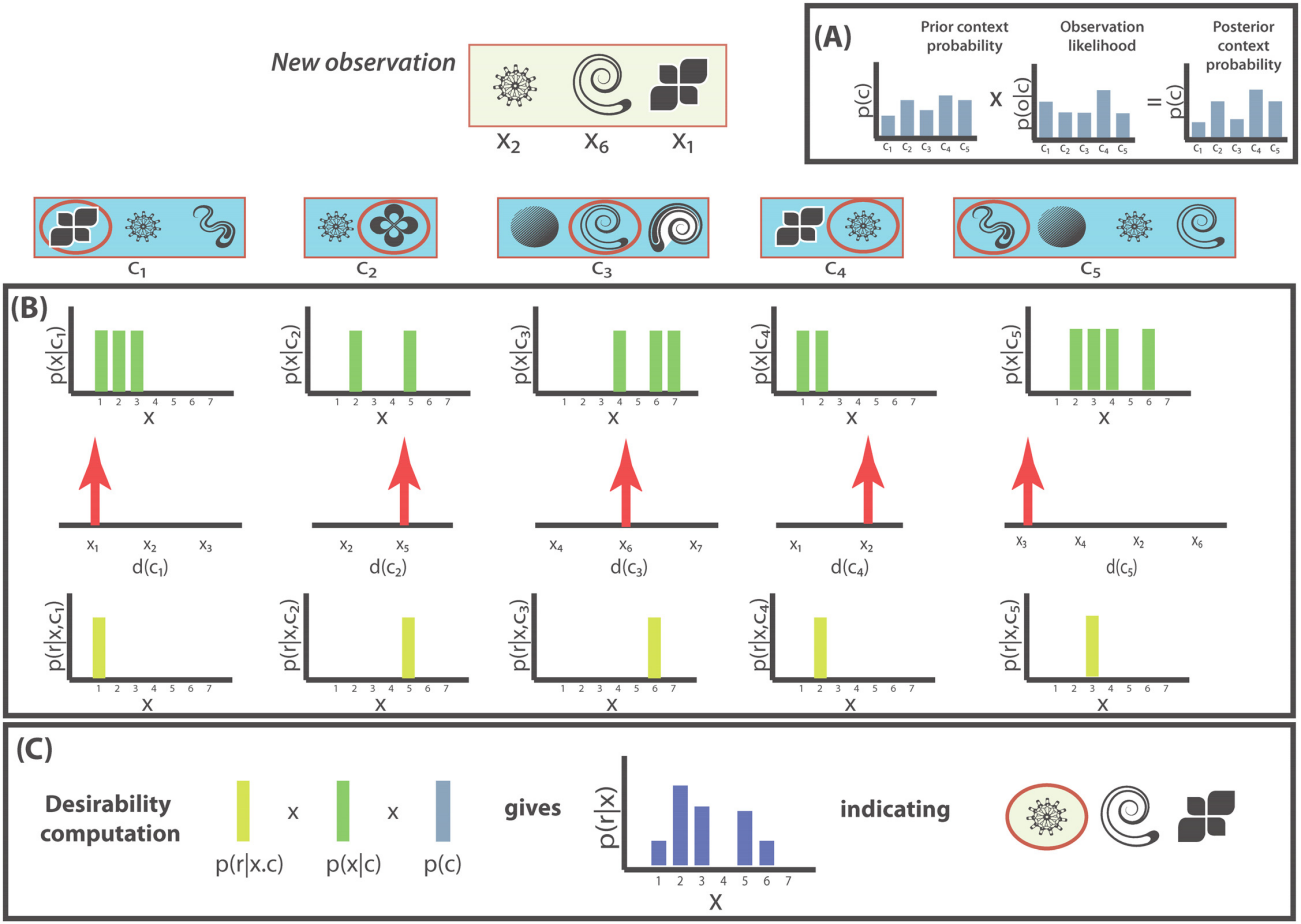
The relative desirability computation. An observer sees a subset of all possibilities in the world at a given time. The observer **(A)** updates its context probability based on the current observation. Subsequently, **(B)** the observer computes desirability and observation probabilities for the new observation and **(C)** uses these intermediate computations to rationally infer relative desirability values for the new option set.

Our approach shares the intuition of accumulating evidence across prior observations weighted by similarity to the current situation, as illustrated in Fig 2. As we describe in the previous section, our approach assumes that people remember past experiences as a tuple 〈*c*, *x*, *r*〉 representing respectively, the context of a choice, the options available, and whether the considered acceptable in that episode. We posit that preferences are determined by accumulating this evidence across multiple episodes in decision relevant ways. Our specific formalism splits this evidence up into three conditional probabilities, each with intuitive interpretations already anticipated above. They are also given in tabular form in Table 1.

**Table 1:** Empirical probabilities in our theory. The relative desirability of an option across all observed contexts can be interpreted as a combination of three probabilistic contributions. These quantities have typically been empirically opaque, but the increasing proliferation of economic activity in digital contexts, where contexts are clearly demarcated, is making them increasingly more empirically available.

Term	Name	Interpretation
*p*(*r*∣*x*, *c*)	Desirability probability	Probability of possibility *x* being desirable within context *c*
*p*(*x*∣*c*)	Observation probability	Probability of observing possibility *x*, given context *c*
*p*(*c*∣*o*)	Context probability	Probability of recalling context *c*, having seen choice set *o*

Evidence accumulation occurs via Bayesian inference over existing memory samples to yield the probability *p*(*r*∣*x*), viz. the probability that option *x* is likely to be acceptable in a new situation. This quantity serves as our preference representation. Observe that given our probabilistic utility representation *p*(*r*∣*x*, *c*), transfer of this information across contexts can be computed simply via a marginalization over observed contexts,

$$\begin{array}{c} R\left(x\right)=p\left(r|x,o\right)=\frac{\sum_{c}^{C}p(r|x,c)p(x|c)p(c|o)}{\sum_{c}^{C}p\left(x|c\right)p\left(c|o\right)}, \end{array}$$ (1)

The *context* probability *p*(*c*∣*o*) is the posterior distribution across contexts given that we have just seen the option set *o*. The context probability acts as a desirability transfer function—it encodes the contribution of value information gathered in other decision contexts to predicting the relative value of the current options. This posterior distribution can be computed via,

$$\begin{array}{c} p\left(c|o\right)=\frac{\sum_{x}p\left(x|o\right)p\left(x|c\right)p\left(c\right)}{\sum_{c}\sum_{x}p\left(x|o\right)p\left(x|c\right)p\left(c\right)}, \end{array}$$ (2)

where *p*(*c*) is a prior distribution over contexts computed previously through prior exposure to other option sets.

To instantiate Eqs (1) and (2) concretely, it is necessary to define a specific form for the *observation* probability *p*(*x*∣*c*). While multiple mathematical forms can be proposed for this expression, for any form it is expected to take a high value for *c* = *o* and proportionately penalize mismatches in set membership. The probability of the entire option set in the observation *o*, *p*(*o*∣*c*) is computed by combining across *p*(*x*∣*c*) terms (see S1 File for details) restricted to the *x* in *o*.

To outline a decision theory within this framework, we assume observers have a decision history they bring to bear on a new decision instance contained in a set of previous decision contexts *c* ^*t*^ ∈ C, and choice information (*r* ^(*t*)^) associated with each context. This makes the summation in Eq (1) a summation across user history. If we want to choose the option in the current set most likely to be desirable we can compute the probability of options.

$$\begin{array}{c} Desirability\left(x\right)=p\left(x|r^{t}=1,c^{\left(t-1\right)},r^{\left(1:t-1\right)}\right)\propto \frac{\sum_{\tau =1:t}p\left(r^{\tau }|x,c^{\tau }\right)p\left(x|c^{\tau }\right)p\left(c^{\tau }|o\right)}{\sum_{\tau =1:t}p\left(x|c^{\tau }\right)p\left(c^{\tau }|o\right)} \end{array}$$ (3)

to obtain a posterior probability encoding the desirability of different possibilities *x*, while also accounting tractably for the context in which desirability information is obtained at every decision instance. For all options available at the moment of decisions, choices are made by selected the option with the highest posterior belief.

The observation probability *p*(*o*∣*c*) in our account performs the same function as the similarity function *s*(*o*, *c*) in CBDT, except that it is operationalized specifically as a function of option set overlap and hence, is computed element-wise in our account. In both accounts though, this term specifies the extent to which the current choice problem is similar to earlier instances in memory, indexed via contexts in our notation, and via the memory tuple in CBDT. The desirability probability *p*(*r*∣*x*, *c*) can be viewed as a stochastic version of *u*(*r*) tied to a specific interpretation of where value comes from—we count win-loss frequency instead of relying upon a hidden hedonic calculation. Where our account diverges from CBDT fundamentally though is in our additional accounting for the base rates *p*(*c*) with which we expect specific contexts to be active given the current observation. In Bayesian terms, rather than rely entirely on the likelihood probability *p*(*o*∣*c*), we also use a prior *p*(*c*) informed by the history of past observations via recursive Bayesian updates as in Eq (2). This prior reasonably accommodates the intuition that frequently encountered contexts will be more accessible to memory, as well as contain more reliable desirability information. We will see further below that this added representational ability extends our theory’s ability to rationalize a larger set of behaviors than CBDT, including, strikingly, violations of the weak axiom of revealed preferences [12].

## Demonstrations

We have so far showed how to interpret the process of learning what-to-do as a rational act of information accumulation. We now characterize situations where preferences learned under our account become equivalent to traditional economic utility, and how these learned preferences can be used in place of expected utility valuations in risky decisions. We thus recover the basic functionality of standard utility functions without actually assuming psychophysical access to value magnitudes.

We also show that our methodology naturally makes choice sets informative about the value of options, and hence affords simple empirical explanations for a number of context effects observed in human subjects in multiple consumer choice experiments [13].

### When is experience-learned desirability equivalent to utility?

Anyone introducing a new preference theory needs to verify that it provides a valid measure of preferences. A preference relation is a partial order on options induced by a person’s comparative evaluation of the options. A preference function is map from options to numbers that validly represents a preference relation. Kreps [14] showed that it is sufficient to show that a preference function *f* on X has the property that: ∀*x*_1_, *x*_2_ ∈ X, *x*_1_ ≻ *x*_2_ ⇔ *f*(*x*_1_) > *f*(*x*_2_).

Within any fixed context, our measure of relative desirability *R* is a preference function, because for any two options *x*_*i*_, *x*_*j*_ ∈ X in a context *c*

$$\begin{array}{c} x_{i}≻x_{j}\Leftrightarrow R\left(x_{i}\right)>R\left(x_{j}\right). \end{array}$$ (4)

However, this condition is violated for agents with context-sensitive preference relations, since our framework specifically allows for preference reversals across decision contexts, rendering the condition *x* ≻ *y* insufficient to characterize context-dependent preference relations. However, we can determine conditions on an agent’s experiences that are sufficient to guarantee that *R* is a preference function. In particular, we find (see S2 File for proof details) that Eq (4) holds under three conditions on the observer’s history enumerated below. Note that the notation C_*i*\*j*_ denotes the subset of all observed contexts that contain *x*_*i*_ but not *x*_*j*_; similarly, C_*j*\*i*_ denotes the subset of all observed contexts that contain *x*_*i*_ but not *x*_*j*_ and C_*ij*_ denotes the subset of contexts that contain both *x*_*i*_ and *x*_*j*_.

(I) **Context consistency**: ∃*c* ∈ C, *s*.*t*. *x*_*i*_ ≻ *x*_*j*_ ⇒ *x*_*i*_ ≻ *x*_*j*_∀*c* ∈ C_*ij*_, {*x*_*i*_, *x*_*j*_} ∈ C_*ij*_ ⊆ C.
(II) **Transitivity between contexts**: if *x*_*i*_ ≻ *x*_*j*_ in *c*_1_ and *x*_*j*_ ≻ *x*_*k*_ in *c*_2_, ∀*c* ∈ C, *x*_*i*_ ≻ *x*_*k*_.
(III) **Symmetry in context observability**: ∀*x*_*i*_, *x*_*j*_ ∈ X, ∣C_*i*\*j*_∣ = ∣C_*j*\*i*_∣.

Condition **(I)** says that option preferences are invariant to context, and condition **(II)** is a standard transitivity requirement on preference orders. Condition **(III)** says that the *number* of contexts in the observer’s history that include *x*_*i*_ but exclude *x*_*j*_ will be matched by an equal number of contexts that include *x*_*j*_ but exclude *x*_*i*_. Of these three assumptions, **(I)** and **(II)** simply define a stable preference relation across contexts and find exact counterparts in the completeness and transitivity assumptions necessary for representing preferences using ordinal utility functions. We note that our proof does not require us to assume continuity or the equivalent Archimedean property to encode preferences, as required in ordinal utility definitions. This is because the continuity assumption is required as a technical condition in mapping a discrete mathematical object (a preference relation) to a continuous utility function. Since relative desirability is defined constructively on *Q* ⊆ ℚ, ∣*Q*∣ < ∞, a continuity assumption is not needed.

Axiom **(III)**, the only additional assumption we require, while stringent, actually uncovers precisely the types of assumptions that differentiate economic analysis from real-world preferences. For instance, two cases in which assumption **(III)** will naturally hold are (i) the typical microeconomics setup where all options in the consumption set are considered available at all times and (ii) the typical experimental economics setup not directed towards testing context and other experience-dependent effects, wherein subjects are given equal exposure to all items. In general, such settings, to the extent that they ask subjects to choose between monetary options, also satisfy conditions **(I)** and **(II)**, given that subjects have a long history of learning to pick larger numbers when associated with money.

Thus, we see that the two most prominent settings in which rational choice modeling is subjected to criticism are, in fact, situations where economic rationality might actually be well-suited for modeling preferences. As soon as we step away from the controlled lab setup, or introduce the possibility of skewed observation possibilities for various options within the experimental setup, we lose the ability to meaningfully describe choice behavior in terms of static relative preferences. More importantly, however, our representation result shows that *it is possible for agents that cannot actually assign absolute values to options to still be able to behave as if maximizing stable relative preferences*.

This finding is congruent with proposals from [15] pointing out that human choice behavior evinces ‘coherent arbitrariness’, viz. humans are very bad at finding the value of anything, suggesting that they do not have any stable measures of value, but once they have estimated some scalar measure of value, they update it responding to changes in the world as if they are maximizing stable relative preferences. Agents following our model of preference learning will behave similarly if the conditions we describe are satisfied; but their initial value estimates will not be arbitrary—they will be learned from past experience. Since experiences are fundamentally idiosyncratic though, the resultant value judgments will appear to be arbitrary.

Our representation result also potentially explains the success of discrete choice modeling in explaining micro-economic human behavior. Since microeconomic discrete choice modeling begins with the premise that all options are always available [16], it axiomatically restricts itself to testing situations which fall within the ambit of assumption **(III)**. There is, therefore, little incentive to design microeconomics experiments or hypotheses that assume differential option availability, resulting in consistent elicitation of data confirming the existence of stable, consistent preferences, with the occasional deviation from transitivity, independence etc. handled by fine-tuning the axiom set to explain anomalies on a case by case basis. While our theory’s prediction—that humans confronted with complete option sets, in the presence of two additional assumptions on their experience history, will always yield rationalizable relative preferences, might seem like epistemic closure to some, it is also possible to interpret this result positively as demonstrating, contingent on the truth of our (falsifiable) value inference theory, that *the epistemic assumptions of economic rationality are, by and large, justified by the subject matter economics examines*.

The novel methodological contribution of the representation result lies in the ability it now gives us to characterize precisely the circumstances wherein inductive economic actors will resemble *homo economicus* at the individual level. Whether deviations from economic rationality arising out of differences in exposure to various options will disappear in competitive market settings is a fascinating open question. Intuitively, if option exposure is a function of structural asymmetries within the market structure that affects one of the three conditions outlined above, (e.g. low income participants are not exposed to luxury goods), we expect deviations from economically rational equilibria to systematically prevail.

Additionally, this result also yields the possibility of quantitatively bounding the *extent* of deviations from economically rational expectations for representative agents in a microeconomic model. Either of our inductive replacements for the deductive completeness and transitivity assumptions allow can be weakened by permitting, for instance *n* violations out of *N* total samples. While settings where *n* ≪ *N* will converge to traditional rational expectations, it also becomes possible to empirically identify *α*-complete and/or *β*-transitive preferences, where *α* = *n*/*N*, *β* = *n*′/*N*. Thus, outlining inductive rationality as a principled generalization of economic rationality renders an erstwhile binary separation between economically rational and irrational preferences into a continuum of quasi-rational preferences, amenable to formal analysis.

Finally, it is instructive to compare the axiomatization we have obtained to that proposed by [17] for the case-repetition version of their case-based decision theory, whose formal setup is identical to ours. Of the five axioms they need to rationalize their proposed theory, the first, order, is identical to assumptions **(I)** and **(II)** in ours. The second, combination, is satisfied by the basic logic of evidence accumulation enforced by Cox’s axioms that underpin our probabilistic account. The third is the Archimedean property that, as we describe above, our inductive account does not need to remain formally coherent. Likewise with the fourth diversity assumption, which entails the presence of all relevant pairwise comparisons in subjective history. Since our account is inductive, our framework is resilient to the presence of Knightian uncertainty—absence of relevant comparisons simply sets the corresponding evidence to zero. The fifth assumption, case independence of desirability, simply enforces the binary nature of the desirability variable *r*, and is aligned with the way we have defined it.

Thus, we see that a context-sensitive version of ordering assumptions can convert our account to a case based decision theory. Thus, not only can our account rationalize preferences outside the ambit of CBDT (by relaxing the ordering assumptions above), even within it, it offers the advantage of specifying not just the causal relationships between history, contexts, and preferences, but also the computations by which these preferences are to be learned.

### Explaining preference reversals in context effects

Since the key motivating feature of our theory is that options in the world are seen in subsets, and that which subset of options is seen is itself informative about the preferences that will be assigned within these subsets, an immediate application suggests itself in the form of explaining a number of ‘context effects’ described in the behavioral economics literature [13].

The term *context effects* refers to systematic reversals of preferences between options brought about by changing only the menu of options presented to subjects, as in the case of Luce’s picky diner. Consider for example Luce’s classic thought experiment of a diner who changes his choice of dinner item from salmon to steak when he learns that frog legs are also on the menu [18]. Intuitively, the presence of frog legs on the menu informs the diner’s inference of the sophistication of the chef’s repertoire, which causes him to change his mind. Inferring the quality of choice options through inferring what context in the world is currently active is a great example of the use of auxiliary information in preference formation in an empirically testable manner. Such behavior is difficult to reconcile with any axiomatic formulation of preferences that maintain that subjects hold stable option-specific preferences. Not only does such *menu-dependence* violate the weak axiom of revealed preferences, it also violates Sen’s simpler *α* condition. As such, it calls into question the possibility of establishing binary choice functions over known options, which is a critical component for any version of rational choice theory [10].

This example maps very easily into our framework of choice selection, wherein the diner’s partial menu observations *o*_1_ = {steak, salmon} and *o*_2_ = {steak, salmon, frog legs} are associated with two separate contexts *c*_1_ and *c*_2_ of observing the menu X. Then, by the problem statement, *p*(*r*∣salmon, *c*_1_) > *p*(*r*∣steak, *c*_1_), while *p*(*r*∣salmon, *c*_2_) < *p*(*r*∣steak, *c*_2_). For the purposes of this demonstration, let us assume these probability pairs, obtained through the diner’s past experiences in restaurants is {0.7, 0.3} and {0.3, 0.7} respectively. Now, when the waiter first offers the diner a choice between steak or salmon, the diner computes relative desirabilities using Eq (1), where the only context for the observation is *c*_1_, implicated via the observation {salmon, steak}. Hence, the relative desirabilities of steak and salmon are computed over a single context, and are simply *R*(salmon) = 0.7, *R*(steak) = 0.3. When the diner is next presented with the possibility of ordering frog legs, he now has two possible contexts to evaluate the desirability of his menu options: implicated by observations of {salmon, steak} and {salmon, steak, frog legs}. Based on his history of experience with both contexts, the diner will have some posterior belief *p*(*c*) = {*p*, 1 − *p*} on the two contexts. Then, the relative desirability of salmon, after having observed frog legs on the menu can be calculated using Eq (1) as,

$$\begin{array}{ccc} R(\text{salmon}) & = & \frac{p\left(r|\text{salmon},c_{1}\right)p\left(\text{salmon}|c_{1}\right)p\left(c_{1}\right)+p\left(r|\text{salmon},c_{2}\right)p\left(\text{salmon}|c_{2}\right)p\left(c_{2}\right)}{p\left(\text{salmon}|c_{1}\right)p\left(c_{1}\right)+p\left(\text{salmon}|c_{2}\right)p\left(c_{2}\right)},\\ & = & \frac{0.7\times 1\times p+0.3\times 1\times (1-p)}{1\times p+1\times (1-p)}=0.7p+0.3\left(1-p\right). \end{array}$$

Similarly, we obtain *R*(steak) = 0.3*p* + 0.7(1 − *p*). Clearly, for 1 − *p* > *p*, *R*(steak) > *R*(salmon), and the diner would be rational in switching his preference. Thus, through our inferential machinery, we retrieve the anecdotal explanation for the diner’s behavior: if he believes that he is more likely to be in a good restaurant than not (with probability (1 − *p*) > 1/2), he will prefer steak.

A number of similar preference reversals have been empirically demonstrated, and are generally referred to as *context* effects [19]. The prevalence of such context effects wounds the rational choice program directly and deeply. If *a* ≻ *b* from within the choice set {*a*, *b*}, but *b* ≻ *a* from within the option set {*a*, *b*, *c*}, then WARP is directly violated, since these two choices are clearly inconsistent. Further, such preference reversals cannot be rationalized using monotone distortions of underlying utility and/or probability representations, as afforded by various generalized utility theories [13], including CBDT [11].

In this section, we show how our theory explains context effects. These are generally enumerated as attraction, similarity, comparison and reference point effects [20]; in each case, a subject reverses his previous preference due to the introduction of a new option into the choice set. Each of these effects can be explained as a rational choice given the preference relationships assumed as background for the effect. Interestingly, all of our explanations are variants of the frog legs example we discuss above [18]. Table 2, provides a compact description of the effects and the background assumptions in a common notation system.

**Table 2:** A unified description of context effects. ≻ indicates stochastic preference for one item over another. Options ⇒_*c*_ relation, indicates that options in a context *c* produce a preference relation. ≻^(−)^ indicates that the preference in question is stochastically weaker than before.

Effect name	Background Assumptions	Effect
Frog legs	{*X*, *Y*} ⇒_*c*_1__ *X* ≻ *Y*, {*X*, *Y*, *Z*} ⇒_*c*_2__ *Y* ≻ *X*	*Y* ≻ *X*
Similarity	{*X*, *Y*} ⇒_*c*_1__ *X* ≻ *Y*, {*X*, *Z*} ⇒_*c*_2__ *X* ∼ *Z*	*Y* ≻ *X*
Attraction	{*X*, *Y*} ⇒_*c*_1__ *X* ∼ *Y*, {*X*, *Z*} ⇒_*c*_2__ *X* ≻ *Y*	*X* ≻ *Y*
Reference point	{*X*, *Y*} ⇒_*c*_1__ *X* ≻ *Y*, {*X*, *Z*} ⇒_*c*_2__ *Z* ≻ *X*	*X* ≻^(−)^ *Y*
Compromise	{*X*, *Y*} ⇒_*c*_1__ *X* ≻ *Y*, {*Y*, *Z*} ⇒_*c*_2__ *Z* ≻ *Y*	*Y* ≻ *X*, *Z*

**Fig 3:**
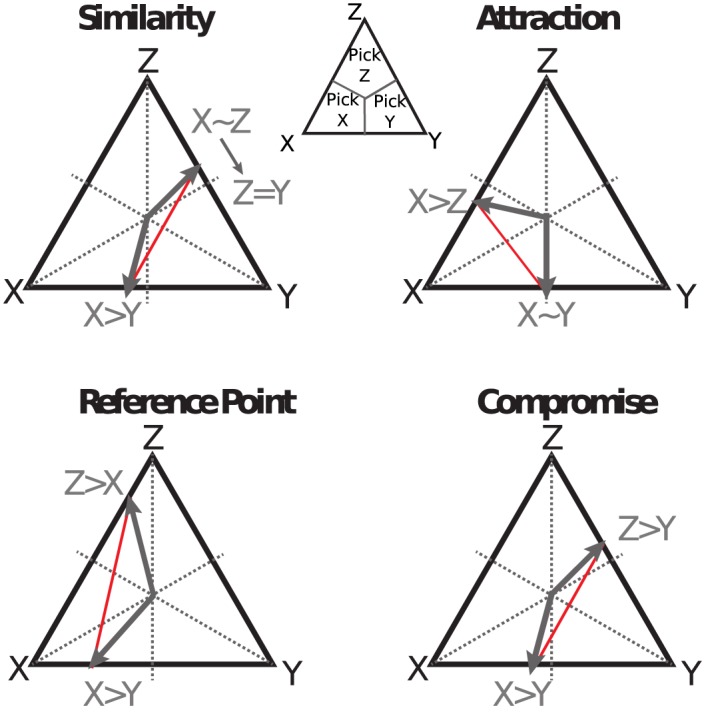
Graphical representation of context effects within our framework. In each triangular preference simplex, as the reference diagram shows, preferences are represented stochastically, such that points closer to vertex *i* represent a relative preference for option *i*. The bigger the distance from the centroid of the triangle, the stronger the preference. For each effect, we encode the background assumptions for the effect, shown in gray, referencing Table 2. Value inference produces a convex combination of these background preferences (all points on the red lines are possible results, depending on the weights used), qualitatively capturing the effect in each case.

Although these effects are often described as distinctive phenomena, we can map them all into a particular form of contextual preference, as described in Table 2. In each of the following demonstrations, an *inductively rational* observer will select the option that has a greater relative desirability value, which, in turn, is computed using Eq (1). Critically, this equation is a convex combination of relative value functions across contexts. In each of the problems pairwise preference information is introduced from more than one context which are combined to predict preferences for a novel option set. Following our table, each of the effects can be graphically illustrated as a convex combination, shown in Fig 3. We provide detailed calculations of the convex combination for each of the effects in S3 File.

#### Frog Legs

In the classic frog legs example [18], where a diner, upon learning that a restaurant offers frog legs as a special, reverses his original preference to order salmon in favor of steak, the reversal in preferences is anecdotally explained by the diner originally forming a low opinion of the restaurant’s chef, given the paucity of choices on the menu, deciding to pick the safe salmon over a possibly burnt steak. However, the waiter’s presenting frog legs as the daily special suddenly raises the diner’s opinion of the chef’s abilities, causing him to favor steak. In our approach, this explanation is simple to formalize. Viewing menu *o*_1_ = {steak, salmon}, the patron believes himself in a context *c*_1_ where salmon is preferred to steak: *p*(*r*∣salmon, *c*_1_) > *p*(*r*∣steak, *c*_1_). However, viewing menu *o*_2_ = {steak, salmon, frog legs}, the patron believes himself in a context *c*_2_ where steak is preferred to salmon: *p*(*r*∣steak, *c*_2_) > *p*(*r*∣salmon, *c*_2_).

#### Attraction Effect

In the attraction effect, we are given a set of choices, {X, Y}, roughly equivalent in preference. When a third option *Z* (the “decoy”) is introduced that is similar in attributes but strongly inferior to *X* on some dimension like price or quality, the subject’s preference is attracted to option *X*. Viewing the option set *o*_1_ = {X, Y}, the patron believes himself in a context *c*_1_ where *X* and *Y* are equivalent: *p*(*r*∣X, *c*_1_) ≈ *p*(*r*∣Y, *c*_1_). The decoy comparison *o*_2_ = {X, Z}, provides a context *c*_2_ where X is preferred: *p*(*r*∣X, *c*_2_) > *p*(*r*∣Z, *c*_2_). When the decoy is added, preferences for {X, Y, Z} are inferred by combining preferences across contexts *c*_1_ and *c*_2_, which increases preferences for *X*. A similar inference predicts results data on human children and capuchin monkeys by [21]. Children were offered stickers and the monkeys M & M’s, until 3 colors were found that were equally preferred, like green red and blue. After a choice between two, say green is chosen over red, preferences decreased for the non-selected item, so the subsequent choices between red and blue showed a boost for blue. While Egan et al regard their findings as evidence of irrational cognitive dissonance, the results are predicted in our approach as an (un)attraction effect.

#### Similarity Effect

In the similarity effect, there is an initial weak preference for *X* relative to Y, *p*(*r*∣X, *c*_1_) > *p*(*r*∣Y, *c*_1_). When a third option *Z* is introduced that is similar to *X* in attributes, preference for Y is boosted. In our approach, adding a similar item essentially means adding an item *Z* such that *p*(*c*∣*x*) ≈ *p*(*c*∣*z*)—items are similar if they invoke the same set of contextual associations. Preference reduction arises if the observer does not possess a choice history for the new item, which is the case in the classic similarity effect descriptions. The addition of the new option expands the number of memory samples that are considered available, without expanding the number of samples wherein *X* is acceptable. This naturally reduces the relative preference for X, causing a preference reversal.

#### Reference Point

The reference point effect has been used to explain many divergent sets of phenomena in the behavioral economics literature. The effect is characterized by a change in value of an item based on the context it appears in. In the effect, items have attributes with natural preference ordering like amount of pain or money. In our account, preferences for an item are changed by introducing additional items with higher or lower attribute values. Menu-dependent preference shifts are almost axiomatic given our setup.

Vlaev et al [22] have provided a compelling experimental demonstration of such menu-dependent preferences: showing that the money people are willing to pay to avoid shocks of varying intensity depends on the range of intensities in the choice set. To avoid shocks of three different intensities, low, medium and high, subjects had to pay money. The researchers found that subjects consistently paid more money to avoid the larger shocks in their recent history. In two sets of experiments, one where low shocks were mixed with medium shocks and one where medium shocks were mixed with higher ones, it was found that subjects paid much more money to buy out of medium shocks in the first condition than the second. Essentially, their evaluation of the undesirability of a particular magnitude of pain was contingent, not on the absolute magnitude of pain they were attempting to avoid, but on the set of pain options.

The inductive explanation for preference reversals clearly satisfies Occam’s Razor—the reversal effects have a substantial literature treating them as independent phenomena, with idiosyncratic explanations. After converting the effects into background desirability assumptions, explanations of each effect emerges graphically as a compromise between pairwise preferences that are assumed in the problem description. It is important to note, though, that while all effect explanations share the convex combination of preferences as their underlying mechanism, it is differences introduced via the background assumptions that actually make the effects hold. Thus, to be precise, the effects are actually explained by the assimilation of preference evidence within constraints imposed by their respective background assumptions. Our theory provides a formal container for the evidence accumulation to proceed normatively, not the explanations themselves.

The only caveat additional to the background assumptions in these demonstrations is that there are limits to the magnitude of initial preferences that can be reversed in our explanation of some of the effects—however, this limitation holds for empirical data as well. Preference reversals do not normally occur for very strong initial preferences. Our theory also makes novel and testable predictions about experimental conditions under which the effect will be exacerbated or diminished, which immediately renders our theory testable within existing experimental paradigms designed to quantitatively study context effects, such as the recently introduced value psychophysics approach [23].

While the effects we explain involve generalizing pairwise preferences to preferences among larger sets, many preference effects do not fit this mold, involving biases due to order of exposure or involving buy no-buy preferences rather than explicit item comparisons. However, our approach is better viewed as inferring preference from a sequence of decisions or experiences. Thus we predict effects due to order of presentation or experience, naturally capturing the sequential and dynamic nature of most reference point phenomena. For example, reference point results in [24] and [25] show that the willingness to pay for two cups of ice cream of different sizes depended on the order in which they were presented to subjects.

We have focused here on presenting detailed explanations for the origins of the major categories of context effects; we believe that it is straightforward to extend the general insight obtained from working through these examples towards explaining other consumption set-linked behaviors.

A typical example is the ‘choice overload’ or set-size effect. One of the basic tenets of traditional decision theory is that adding more choices for subjects can never be a bad thing (‘more information is always good’). Multiple studies document violations of this assumption in real-world purchasing behavior [26]. Empirical evidence suggests that subjects are more likely to select an option when it is presented to them in smaller choice sets than in larger ones. Our theory explains set size effects as an extension of the explanation we provide for similarity effects—they arise out of the redistribution of probability mass corresponding to known options to other similar (or related) options. Our theory also predicts that set size will not affect consumers with strong preference for a particular subset of items. They will just as easily pick from amongst this subset standing alone as when it is embedded within a much larger consumption set.

Competing hypotheses that seek to explain within-subject preference reversals under choice set variation are either descriptive and static (e.g. quantum cognition [27]), normative and static, (e.g. extended discrete choice models ([28] provides a recent review), componential context theory [19]) or descriptive and dynamic, (specifically, decision field theory [29]). In contrast, our approach not only takes a dynamic inductive view of value elicitation, it retains a normativity criterion (Bayes rationality) for falsifying observed predictions, a standard that is expected of any *rational* model of decision-making [30].

An approach that is both normative and dynamic in explaining three of the context effects outlined in this section (all expect reference point effects) comes from [26, 31], who agree with our basic idea that value is inferred from the set of available options, but assume that there is some stable *preference* corresponding to each option, which is progressively uncovered when more options are observed. The critical difference between this idea (which we call ‘information scarcity’) and ours is that whereas proponents of this view believe that preference reversals arise due to increase in information about the stable, context-free value of options, we believe that value is contextual and labile.

Thus, a testable difference between these two approaches is that we predict that, subsequent to a preference reversal trial, when subjects encounter the original two object choice again, they will again revert to their original choice set specific preference. Our theory predicts that context effects can be elicited over and over, whereas according to the information scarcity proposal, given the same option sets again, context-based preference reversals will not arise. Existing empirical evidence demonstrating repeated context effect elicitations both in experimental and market settings [26] weighs in favor of our prediction.

Finally, while the alternative theories we have discussed above can potentially explain some or all of the context effects we have discussed, they make intrinsically economic assumptions and hence, cannot purport to explain the existence of context effects in domains outside economic decision-making, e.g. attraction effects seen in hummingbirds [32], compromise effects seen in the amoeba *physarum polycephalum* [33] and perceptual recognition tasks in humans [34]. These studies demonstrate that context effects cannot be simply explained through invoking simple economic ideas, they are in fact deeply rooted in the information processing systems of organisms and hence, require an information-processing explanation of the type that we provide.

## Discussion

### 

#### Implications for economic theory

“It is clear that economics rests on some sort of implicit psychology. The question has been whether this implicit psychology is good psychology or bad psychology” [35]. By developing a psychological theory of preference formation that emits economically rational preferences as a special case, we have assisted in clarifying the boundaries between the two disciplines and presented evidence showing that the implicit psychology underlying economics is good psychology assuming certain conditions apply on the history of subjects’ experience. We have also rationalized a number of behavioral economics critiques of standard theory as deviations from the ideal conditions needed for the implicit psychology of economics to be a good match for the phenomena.

But how to integrate this theory of how preferences are formed with economic theories more generally? It is tempting to argue that clarifying the dynamics of preference evolution at the level of individual households immediately provides a new class of adaptive microfoundations—one sensitive to observed individual differences in transaction history. But whether such microfoundations are necessary at all is not entirely clear.

Debates about the value of microfoundations themselves often devolve into arguments about how much detail a particular model needs in order to be useful. The answer, usually, is ‘It depends.’ As a consequence, microeconomists usually prefer to deal with psychological insights on a case-by-case basis, incorporating stylized assumptions about the behavior of representative agents that appear justified within the particular market structure they are investigating.

Such psychological agnosticism leaves microeconomic models susceptible to the Lucas critique—they can measure econometric variables, but have no theory to rely on to predict how these variables will evolve in response to policy changes and other exogenous inputs. The common epistemological workaround—rational expectations—assuming that agents are foresighted and take future changes into account in their current behavior, avoids this difficulty, but grants agents near-magical information-gathering abilities that they do not possess in the real world. Further modifications to the rational expectations program have added sensitivity to information asymmetries and costs back into the picture, resulting in the sorts of microeconomic agents that are currently considered satisfactory.

Viewed from a psychological standpoint, by eschewing adaptive expectations, the microfoundations project is severely harmed at its very inception. Multiple lines of empirical evidence converge on the view that real people are more reactive than foresighted. Preferring rational over adaptive expectations modeling forces the microfoundations project to come up with ways of describing behavior that is disconnected from this simple reality, causing some to consider it fundamentally unsatisfactory [36].

We present a way out. Given the success of our theory in explaining a variety of economic behaviors as simple information updating, it seems likely that any adaptive expectation based microfoundations should engage with the idea that preferences evolve given exposure to choice sets in the manner we have shown. Thus, we believe this work can serve as a mathematical basis for the development of a new generation of adaptive microfounded economic models. Given increased access to transaction histories and other economic behaviors, particularly from e-commerce portals, it is easy to be optimistic about the possibility of deeper and more data-driven microfoundations informing economic policy in years to come.

An important step in such theoretical development is the re-introduction of numerical magnitudes as a source of information during preference learning. Recall that we have explicitly renounced using magnitude-based representations of value, reflecting psychological and behavioral economics findings that people do not appear to have stable internal representations of value [3]. However, external magnitude labels do convey information about what to do in the world—people are much more likely to prefer an option with an expected value of $20000 than one with an expected value of $2. In recent work, we have formally extended our current framework to permit preference learning while incorporating auxiliary information from magnitude labels [37]. A closer integration of this augmented framework with economic theory and applications remains an important target for future work.

#### Related work

The inadequacy of utility maximization as a theory of human behavior has long been noted. While economists have largely remained agnostic to the behavioral implications of utility theory, researchers in other allied disciplines have sought alternative explanations. Since [38], decision-theorists have sought to develop utility-free random decision rule (RDR) models that seek to explain choice behavior procedurally using various heuristics. Prominent examples of such heuristics include EBA [39] and lexicographic heuristics [40]. While the biases and heuristics research program has found significant success in explaining behavior in particular ecological niches (e.g. specific marketing applications), it has proved very difficult to generalize such models, let alone make them rationalizable.

Closer to mainstream economics, the research programs of [1] and [41] have attempted to discover hypotheses that constrain the types of strategies that subjects might use to construct preferences when needed. While these efforts did not lead to formal theories, they have introduced to the economics community a key idea that we formalize in our work—that preferences may be generated *de novo* at the time of decisions. We have already discussed the conceptual similarity our proposal shares with the Gilboa-Schmeidler case-based decision theory [11]. As we note above, our approach can be viewed in some sense as an extension of CBDT—adding the ability to learn case similarity from exposure history and removing the necessity for decision-makers to store decision *utility* in memory.

The idea that a person’s history of experience informs their future preferences is canonical in psychology and cognitive science. Beginning with Pavlov’s discovery of classical conditioning [6], researchers have found numerous ways in which an animal’s preference for particular items can be reinforced, attenuated and/or changed in valence using simple experimental manipulations [42]. While meticulous, such research has generally not interacted well with economics, since the emphasis has been on probing the associative nature of human learning rather than informing models of preference formation. More directly relevant is recent research by Palmer & Schloss [43] who demonstrate empirically how humans’ history of object preferences strongly predict their preferences for basic colors, and odors (unpublished, private communication). Such results provide promising supporting evidence for our theory.

We are also by no means the first to observe that the frequency of observing (binarized) gains appears to be an equivalent, and sometimes better, predictor of choice behavior than expected value. Similar results are repeatedly documented by neuroeconomics researchers studying the similar Iowa Gambling Task [44] and its derivative Soochow Gambling task [45]. In these studies (see [46] for a review), researchers find that subjects consistently prefer options with lower expected value but higher gain-loss frequency. This validates the hypothesis that the frequency of observing gains is a better predictor of choice behavior than expected value [45], a conclusion that is entirely concordant with our theory. The proposal that pairwise comparisons lie at the heart of preferences can be traced as far back as Thurston’s seminal work.

It is interesting to note that several models of preference formation that closely resemble our proposal come from the politicial science community. Since the objects of interest to this community are closer to binary point objects than the consumption-centric containers of utility in economic modeling, political science researchers have also developed models of preference learning that attempt to learn preferences from binary comparisons (see [47] for a comprehensive review).

While all the proposals above engage to varying degrees with the idea that preferences are changeable, none can both dynamically learn preferences and rationalize them. Earlier models that have attempted to learn preferences have had to restrict themselves to learning only economically rational preferences describable by utility functions, as in the case of preference learning techniques in artificial intelligence [48–50] and in Camerer’s experience-weighted-attraction scheme [51]. Thus, to the best of our knowledge, our work constitutes the first rational theory of preference formation.

#### Limitations of this approach

While our theory can explain a broad class of preference-related effects, it fails as a universal theory of preference formation for a fundamental reason. Namely, our approach is dependent on the ability of subjects to accurately estimate event probabilities, an assumption which is known to be false. Probability assessments are clearly distorted in ways that are well-documented, but for causes that still remain unknown (see e.g. [52] for a recent review). Since our theory cannot account for distortions in probability judgment, it cannot explain deviations from economic rationality that arise through them, e.g. the pattern of risk aversion and seeking demonstrated in prospect theory. A sample demonstration showing the inability of our theory to explain the Allais paradox is described in S4 File.

Since our theory is modular with respect to event probabilities, it lets us add probability distortions in by hand, as we do to explain empirical lottery data. However, such *ad hoc* combination with prospect theory is theoretically unsatisfactory, and suggests that a more rigorous investigation of subjective probability is necessary. In the interim, however, understanding the limitations that probability inference places upon our theory allows us to demarcate the class of behaviors for which it will yield reasonable predictions. This understanding can be formalized using the Cox formulation of Bayesian probability. Consider the set Q of all possible propositions *q* about the world, a belief function *p* : *Q* → [0, 1] that measures the likelihood of a proposition being true, and the notation *p*(*q*_1_∣*q*_2_) to reflect belief that *q*_1_ is true given we know *q*_2_ is true. Then, for *p* to be a Bayesian probability, three operations should be possible which we axiomatize as follows,

1. **(Ordering)** ∃ > ∣*p*(*q*_1_) > *p*(*q*_2_) ⇔ *q*_1_ likelier than *q*_2_
2. **(Negation)** ∃*f* ∣*p*(¬*q*) = *f*(*b*(*q*))
3. **(Association)** ∃*g* ∣*p*(*q*_1_ ∧ *q*_2_) = *g*(*p*(*q*_1_), *p*(*q*_2_∣*q*_1_))

While these, the Cox axioms, appear quite rudimentary, they are known to be violated frequently by human observers. For instance, the ordering axiom is violated by subjects showing distorted probability judgments for low-probability options in prospect theory experiments [53]. Negation is violated by observers evincing subadditive biases of generalization [54], whereas association is clearly violated by observers exhibiting disjunction effects [55]. Such probability estimation biases, therefore, fall outside the ambit of rationalizable choices attributable to our theory.

Our theory also assumes that agents can easily perform the Bayesian computations necessary to obtain relative desirabilty information from the world. This would mean that an agent making a familiar choice for the 2000^*th*^ time, say deciding which route to take to get to ofice, would have to draw upon all 1999 past experiences before making a decision. Such a mechanism would also predict that familiar choices require *more* cognitive effort than novel ones. That this is not the case is both intuitive and supported by empirical data on the effects of habituation on choice response times [56]. In practice, it is likely that only a limited subset of experiences is recalled from memory during value inference. Interestingly, prior research has implicated memory recall as a potential candidate for explaining the origins of probability distortions [53, 57]. Therefore, understanding how the memory recall process works may also yield an explanation for how humans actually compute probabilities.

Finally, our current framework also fails to account for temporal factors that are known to play important roles in preference dynamics, e.g. intertemporal discounting. Discovering how such temporal factors interact with our theory constitutes an important direction for future research. Operationalizing our Bayesian causal theory using algorithms that better match the mechanistic processes that humans actually use to generate subjective probabilities will likely result in further improvements in explanatory power [57].

Thus, we can identify at least three ways in which peoples’ decisions will deviate from the predictions of our theory. One, because of cognitive limitations leading to probability distortions; two, because of cognitive processing of temporal expectations, leading to inter-temporal inconsistency, and three, because of social influences that cause people to behave in ways not congruent with their personal preference. This last source of deviation can be integrated into our model *post hoc*—once someone has made a choice based on social influence, their choice history will predispose them to do so again via our theory. However, the other two cannot be integrated thusly, and will require further research to resolve.

#### Conclusion

Assuming that people maximize expected utility is a statement about the information that we believe observers retain about their choices. It is, of course, true that the economic assumption of utility maximization is not a claim about the psychological representation of value that human observers are likely to possess. However, committing to such a representation enforces the omission of preferences incompatible with utility maximization accounts, e.g. preference reversals, from economic explanations. One could argue that losing out on representing inconsistent choices is not necessarily a big loss. The more critical problem with using a utility-based representation is practical—it renders the process by which observers arrive at these utility values in the first place opaque, which means that any effects of individual differences due to experience have to be induced artificially, not determined econometrically.

By replacing utility maximization with desirability inference, we accomplish three goals. One, we solve the representation problems endemic to treating information about what-to-do in a privileged manner in computational terms [4]. Two, we expand the range of preferences that can be formally rationalized in economic modeling [13]. Three, we give a practical way of learning preferences from choice histories, such that both preferences and individual differences can be learned together. While this naturally requires more data than just revealed preferences, this is less of a concern in today’s digital age than it would have been even five years ago, and should be vanishingly less so going forward.

By developing a workable theory of psychological rationality and situating economic rationality within it both theoretically and empirically, our work bridges the theoretical divide between economics and psychology. Replacing option-specific utilities with learned relative preferences elicited from behavior history is expected to yield explanations of valuation in both microeconomics and psychology that hew closer to actual human behavior.

*Wealth—any income that is at least one hundred dollars more a year than the income of one’s wife’s sister’s husband*.

H L Mencken

## Supporting Information

S1 File Details of the observation probability definition. (PDF)

S2 File Proof of the representation result. (PDF)

S3 File Context effect demonstrations. (PDF)

S4 File The Allais paradox is still paradoxical. (PDF)
