# Supplementary material for: Learning What to Want: Context-Sensitive Preference Learning
Source: PLoS One. 2015 Oct 23;10(10):e0141129. doi: 10.1371/journal.pone.0141129 (PMC4619741; doi:10.1371/journal.pone.0141129)
Supplement: S1 File — (PDF) [file pone.0141129.s001.pdf]

Details of the observation probability definition

Defining the observation probability $p(x|c)$ in terms of element-wise mismatches between the observation subset and the context subset of world possibilities requires us to maintain two indices over either type of subset. We use y^t to denote an indicator function on $\mathcal{X}$ encoding the possibilities observed as $o^{(t)}$,

$$y^t(x) = \sum_{i \in o^{(t)}} \delta(x - i).$$

Similarly, we index contexts with an indicator function z on $\mathcal{X}$, so that for context $c^{(t)}$,

$$z^t(x) = \sum_{i \in c^{(t)}} \delta(x - i).$$

Given this indexing, we can denote the element-wise mismatch probability as $p(\neg y_i^t | z_i^t)$. Since $p(x_i|c^{(t)}) = 1 - p(\neg y_i^t | z_i^t)$, we can use these element-wise probabilities to compute the likelihood of any particular observation $o^{(t)}$ as,

$$p(o^{(t)}|c^{(t)}) = 1 - p\left(\bigcup_i^{|o^{(t)}|} \{\neg y_i^t\} | \bigcup_i^{|c^{(t)}|} \{z_i^t\}\right) = 1 - \beta \sum_i^{|\mathcal{X}|} p(\neg y_i^t | z_i^t), \tag{1}$$

where β is a parameter controlling the magnitude of the penalty imposed for each mismatch observed.

To concretely instantiate our likelihood definition in (1), we define a specific mismatch probability,

$$p(\neg y_i^t | z_i^t) = \frac{1}{|\mathcal{X}|}\left((1 - z_i^t) y_i^t + (1 - y_i^t) z_i^t\right), \tag{2}$$

with $\beta = 1$ for all our demonstrations.
