# Supplementary material for: Learning What to Want: Context-Sensitive Preference Learning
Source: PLoS One. 2015 Oct 23;10(10):e0141129. doi: 10.1371/journal.pone.0141129 (PMC4619741; doi:10.1371/journal.pone.0141129)
Supplement: S2 File — (PDF) [file pone.0141129.s002.pdf]

Proof of representation result

To show that our measure of relative desirability R also completely represents preference information, it is sufficient to show that, for any two possibilities $x_i, x_j \in \mathcal{X}$, and for any context c

$$x_i \succ x_j \Leftrightarrow R(x_i) > R(x_j). \tag{1}$$

Since the existence of preference reversals through context variation destroys the possibility of a stable preference relation, we begin by restricting our analysis to preferences that satisfy a **context consistency** requirement,

$$\exists c \in \mathcal{C}, s.t.\ x_i \succ x_j \Rightarrow x_i \succ x_j \forall c \in \mathcal{C}_{ij}, \{x_i, x_j\} \in \mathcal{C}_{ij} \subseteq \mathcal{C}. \tag{2}$$

This additional requirement makes the expression of preferences in the context-aware setting epistemologically equivalent to the standard characterization of binary preference, since an observer insensitive to context will simply find that $x_i \succ x_j$ whenever the two possibilities are observed together. To completely characterize a preference relation over $\mathcal{X}$, however, simply specifying consistent binary preferences is insufficient. Analogous to the regular concept of transitivity, we further assume the existence of **transitivity between contexts**, such that,

$$\text{if } x_i \succ x_j \text{ in } c_1 \text{ and } x_j \succ x_k \text{ in } c_2, \forall c \in \mathcal{C}, x_i \succ x_k, \tag{3}$$

thereby introducing a sense of preference order across observable contexts.

Now, consider that for any pair of possibilities $\{x_i, x_j\} \subseteq \mathcal{X}$, the set of observable contexts can be partitioned as,

$$\mathcal{C} = \mathcal{C}_{\setminus ij} \cup \mathcal{C}_{i\setminus j} \cup \mathcal{C}_{j\setminus i} \cup \mathcal{C}_{ij},$$

with the subscript indices indicating the possibilities from among $\{x_i, x_j\}$ considered feasible, i.e.$p(x|c) = 1$ within that context subset. Let $\mathbb{C} = \{\mathcal{C}_{\setminus ij}, \mathcal{C}_{i\setminus j}, \mathcal{C}_{j\setminus i}, \mathcal{C}_{ij}\}$. Then,

we can expand the desirability definition from the main text (Equation 1) to,

$$R(x) = \frac{\sum_i^{|\mathbb{C}|} \sum_c^{\mathcal{C}^{(i)}} p(r^{(t)}|x,c)p(x|c)p(c)}{\sum_i^{|\mathbb{C}|} \sum_c^{\mathcal{C}^{(i)}} p(x|c)p(c)}, \tag{4}$$

Using our definitions of $p(x|c)$ and $p(r|x,c)$ (see SI: Details of the observation probability), it is straightforward to show that,

$$R(x_i) = \frac{k_i \sum_c^{\mathcal{C}_{i\setminus j}} P(c) + k_{ij} \sum_c^{\mathcal{C}_{ij}} P(c)}{\sum_c^{\mathcal{C}_{i\setminus j}} P(c) + \sum_c^{\mathcal{C}_{ij}} P(c)}, \quad R(x_j) = \frac{k_j \sum_c^{\mathcal{C}_{j\setminus i}} P(c) + k_{ji} \sum_c^{\mathcal{C}_{ij}} P(c)}{\sum_c^{\mathcal{C}_{j\setminus i}} P(c) + \sum_c^{\mathcal{C}_{ij}} P(c)}, \tag{5}$$

since all other contributions disappear due to corresponding entries in $p(x|c)$ being zero. Here, the single indexed k_i counts the number of times possibility x_i was considered the most desirable in contexts including x_i and excluding x_j; k_j being defined symmetrically. The double-indexed k_{ij} counts the number of times x_i is considered the most desirable possibility in contexts where x_j is also believed to be present. Again, k_{ji} is defined symmetrically.

From (5) it should be clear that, in general, differences in the sampling of contexts in an agent's history of observations, measured, for instance, as variations in the size of the context subsets $\mathbb{C}^{(i)}$ will render comparisons between desirability values undecidable.

To see why this must be the case, observe that for any two functions of homologous form to R such that $\frac{\alpha k_i + k_{ij}}{\alpha+1} = \frac{\beta k_j + k_{ji}}{\beta+1} + \theta$, with the k values fixed, it is always possible to find a new $\beta' = \beta\left(1 + \frac{\theta}{k_j} + \frac{\theta}{k_j(\beta+1)}\right) + 1$ that will reverse the inequality.

Hence, to retain consistent preferences, we require an additional condition on the history of contexts that generate our relative desirability measure. Specifically, we assume,

$$\forall x_i, x_j \in \mathcal{X}, |\mathcal{C}_{i\setminus j}| = |\mathcal{C}_{j\setminus i}|, \tag{6}$$

reflecting the intuition that there be no informative reason underlying the partial observability of world possibilities, i.e., partial observability occurs via random subset selection from $\mathcal{X}$. Note that this assumption, by symmetry, also implies

$$\lim_{t\to\infty} p(x|\text{data}) = U(x), \tag{7}$$

$U(\cdot)$ representing the uniform distribution.

Given this, in the infinite data limit, we obtain

$$
\begin{aligned}
p(x|data) &= \sum_{c}^{\mathcal{C}_{i\setminus j}} p(c) + \sum_{c}^{\mathcal{C}_{ij}} p(c) = \sum_{c}^{\mathcal{C}_{j\setminus i}} p(c) + \sum_{c}^{\mathcal{C}_{ij}} p(c) = U(x), \\
\Rightarrow \sum_{c}^{\mathcal{C}_{j\setminus i}} p(c) &= \sum_{c}^{\mathcal{C}_{i\setminus j}} p(c),
\end{aligned}
$$

obviating the necessity of further accounting for the denominators in (5).

It is now quite straightforward to demonstrate both directions of (1). First, assuming the left hand side of (1) immediately sets $k_{ji} = 0$. Further, using symmetry in context observability, k_i can now be interpreted as determining the number of times x_i dominates all other possibilities in $\mathcal{X} \setminus \{x_j\}$; k_j vice versa. By (3) x_i dominates all possibilities that x_j dominates, by (6) the number of observations over which either possibility can dominate is equal and by (7), in the limit of infinite decision samples, they will observe the same alternative possibilities, implying $k_i \geq k_j$. Since $k_{ij} > 0$, we directly have,

$$
\begin{aligned}
k_i \sum_{c}^{\mathcal{C}_{i\setminus j}} p(c) + k_{ij} \sum_{c}^{\mathcal{C}_{ij}} p(c) &\geq k_j \sum_{c}^{\mathcal{C}} p(c), \\
&\Rightarrow R(x_i) > R(x_j).
\end{aligned}
$$

Assuming the RHS of (1) to be true, adopting any reasonable selection rule, e.g. $\max_x R(x)$ proves the converse. Hence, contingent on the three assumptions we have specified above, the relative desirability based decision framework encodes relative preference relations equivalently well as ordinal utility functions.
