# Supplementary material for: Learning What to Want: Context-Sensitive Preference Learning
Source: PLoS One. 2015 Oct 23;10(10):e0141129. doi: 10.1371/journal.pone.0141129 (PMC4619741; doi:10.1371/journal.pone.0141129)
Supplement: S3 File — (PDF) [file pone.0141129.s003.pdf]

Context effect demonstrations

In each of the following demonstrations, an *inductively rational* observer will select the option that has a greater relative desirability value, which, in turn, is computed using Equation **??**. We turn first to Luce's classic thought experiment. In the frog legs example, the reversal in preferences is anecdotally explained by the diner originally forming a low opinion of the restaurant's chef, given the paucity of choices on the menu, deciding to pick the safe salmon over a possibly burnt steak. However, the waiter's presenting frog legs as the daily special suddenly raises the diner's opinion of the chef's abilities, causing him to favor steak.

This intuition maps very easily into our framework of choice selection, wherein the diner's partial menu observations $o_1 = \{\text{steak, salmon}\}$ and $o_2 = \{\text{steak, salmon, frog legs}\}$ are associated with two separate contexts c_1 and c_2 of observing the menu $\mathcal{X}$. Bad experiences related to ordering steak in menus typically observed under context c_1 (interpretable as 'cheap restaurants') may be encoded by defining the vector $m = \{1, 0, 0, 0\}$ for c_1 and good experiences ordering steak off menues observed in context c_2 (interpretable as 'upscale restaurants') as $m = \{0, 1, 0, 0\}$ for c_2.

Then, by definition, $p(r|\text{salmon}, c_1) > p(r|\text{steak}, c_1)$, while $p(r|\text{salmon}, c_2) < p(r|\text{steak}, c_2)$. Assume these probability pairs, obtained through the diner's past experiences in restaurants to be $\{a, 1-a\}$ and $\{1-b, b\}$ respectively. When the waiter first offers the diner a choice between steak or salmon, the diner computes relative desirabilities using (**??**), where the only context for the observation is {salmon, steak}. Hence, the relative desirabilities of steak and salmon are computed over a single context, and are simply $R(\text{salmon}) = 0.7, R(\text{steak}) = 0.3$.

When the diner is next presented with the possibility of ordering frog legs, he now has two possible contexts to evaluate the desirability of his menu options: {salmon, steak} and {salmon, steak, frog legs}. Based on the sequence of his history of experience with both contexts, the diner will have some posterior belief $p(c) = \{p, 1-p\}$ on the two contexts. Then, the relative desirability of salmon, after

having observed frog legs on the menu can be calculated using (**??**) as,

$$R(\text{salmon}) = \frac{p(r|\text{salmon}, c_1)p(\text{salmon}|c_1)p(c_1) + p(r|\text{salmon}, c_2)p(\text{salmon}|c_2)p(c_2)}{p(\text{salmon}|c_1)p(c_1) + p(\text{salmon}|c_2)p(c_2)},$$
$$= \frac{a \times 1 \times p + (1-a) \times 1 \times (1-p)}{1 \times p + 1 \times (1-p)} = ap + (1-a)(1-p).$$

Similarly, we obtain $R(\text{steak}) = (1-b)p + b(1-p)$. Clearly, independent of the values of a and b, if $1-p > p, R(\text{steak}) > R(\text{salmon})$, and the diner would be rational in switching his preference. Thus, through our inferential machinery, we retrieve the anecdotal explanation for the diner's behavior: if he believes that he is more likely to be in a good restaurant (with probability $(1-p)$) than not, he will prefer steak.

Along identical lines, making reasonable assumptions about the contexts of past observations, our decision framework accomodates parsimonious explanations for each of the other effects detailed in Table **??**. In all the cases described below, we assume particular initial levels of relative desirability during calculation. For consistency, across our demonstrations, indifference between options X and Y is taken to imply $p(r|X, c = \{X, Y\}) = p(r|Y, c = \{X, Y\}) = \mathbf{0.5}$. A weak preference for X over Y is taken to imply $p(r|X, c = \{X, Y\}) = \mathbf{0.6}, p(r|Y, c = \{X, Y\}) = \mathbf{0.4}$, and a strong preference for X over Y is taken to imply $p(r|X, c = \{X, Y\}) = \mathbf{0.8}, p(r|Y, c = \{X, Y\}) = \mathbf{0.2}$.

In some cases, we find partitions of relative desirability between options that render a particular demonstration invalid. We will point these out where relevant, and interpret them as theoretical substantiations of the observed fragility of these effects to changes in the relative desirabilities of the options in the initial choice set.

Similarity effect

In the similarity effect, given a choice set XY, a subject (weakly) prefers option X. Now, a third option Z is introduced into the choice set, which is known to be similar to X, and not generally perceived to be clearly superior or inferior to X. In this expanded choice set XYZ, the subject is observed to reverse his preference and select Y.

Assume that the distribution over the observation contexts XY and XZ is $\{p, 1-p\}$ (YZ is never observed, per the standard similarity effect setup) Because of the similarity between X and Z, evidence for the desirability of X is computed over both observations

of X and Z such that,

$$\begin{aligned} R(X) &= \frac{\sum_x^{\{X,Z\}} p(r|x,XY)p(x|XY)p(XY) + \sum_x^{\{X,Z\}} p(r|x,XZ)p(x|XZ)p(XZ)}{\sum_x^{\{X,Z\}} p(x|XY)p(XY) + \sum_x^{\{X,Z\}} p(x|XZ)p(XZ)}, \\ &= \frac{0.6 \times 1 \times p + 0 + 2 \times 0.5 \times 1 \times (1-p)}{1 \times p + 1 \times (1-p) + 1 \times p + 1 \times (1-p)}, \\ &= \mathbf{0.5 - 0.2p}, \end{aligned}$$

while a similar computation for $R(Y)$ yields,

$$\begin{aligned} R(Y) &= \frac{\sum_1^{10} p(r|Y,XY)p(Y|XY)p(XY) + \sum_1^{10} p(r|Y,XZ)p(Y|XZ)p(XZ)}{\sum_1^{10} p(Y|XY)p(XY) + \sum_1^{10} p(Y|XZ)p(XZ)}, \\ &= \frac{0.4 \times 1 \times p + 0 \times 0 \times (1-p)}{1 \times p + 0 \times (1-p)}, \\ &= \mathbf{0.4}, \end{aligned}$$

and $R(Z) = \mathbf{0.5 - 0.5p}$.

For all values of $p > 1/2$, $R(X) < R(Y)$, resulting in a rational preference reversal $Y \succ X$, suggesting that the similarity effect is sensitive to the extent to which the comparison between the similar objects is salient in the choice domain. This dependence has an intuitive explanation. For high values of p, such comparisons will be rare, and cause a preference reversal in the original choice set. However, when the similarity comparison dominates the original choice comparison, the subject generalizes his preference $X \succ Y$ to the new object Z.

In the particular case of the similarity effect, calculation using more extreme values for the original XY preference (e.g. 0.9/0.1 instead of 0.6/0.4) suggests that the similarity effect will not arise in cases where X is strongly preferred to Y, an easily testable prediction from our theory. Further, the inference mechanism allows us to also predict that the similarity effect will return in such cases with the introduction of yet more items Z' similar to X into the choice set, causing the preference for X to be further multiply redistributed.

Attraction effect

In the attraction effect, given a set of choices, {X,Y}, the subject is originally seen to be indifferent between the two options. However, when a third option Z that is similar to,

but strongly inferior to option X is introduced into the choice set, the subject's preference switches to prefer option Y.

In the extended choice set regime, the desirability computation for X yields,

$$\begin{aligned} R(X) &= \frac{p(r|X,XY)p(X|XY)p(XY) + p(r|X,XZ)p(X|XZ)p(XZ)}{p(X|XY)p(XY) + p(X|XZ)p(XZ)}, \\ &= \frac{0.5 \times 1 \times p + 0.8 \times 1 \times (1-p)}{1 \times p + 1 \times (1-p)}, \\ &= \mathbf{0.8 - 0.3p}, \end{aligned}$$

while a similar computation for Y yields,

$$\begin{aligned} R(Y) &= \frac{p(r|Y,XY)p(Y|XY)p(XY) + p(r|Y,XZ)p(Y|XZ)p(XZ)}{p(Y|XY)p(XY) + p(Y|XZ)p(XZ)}, \\ &= \frac{0.5 \times 1 \times p + 0}{1 \times p + 0}, \\ &= \mathbf{0.5}. \end{aligned}$$

p being a probability with non-zero support for both contexts $p \in (0,1)$, implying that $R(X) > R(Y)$ for any value of p, resulting in the establishment of a rational preference $X \succ Y$ in place of the earlier indifference. This conclusion is also expected to hold for any value of XZ preferences s.t $p(r|X,c) > p(r|Z,c)$, which is precisely the condition that the attraction effect requires.

Furthermore, our inferential process in this setup also predicts that an item that is originally equivalent in desirability to another item that is then found to be inferior to a third item in a separate choice set will be subsequently found preferred in the original comparison. Interestingly, this secondary prediction has been verified in human infants and capuchin monkeys by [?]. While Egan et al regard their findings as evidence of irrational cognitive dissonance, the results from their experimental task are clearly interpretable as an (un)attraction effect, and hence, compatible with our explanation.

Reference point effect

The reference point effect has been used to explain many divergent sets of phenomena in the behavioral economics literature. For our demonstration, we restrict ourselves to explaining the results of a particular experiment on human subjects due to Vlaev et

al [?], where subjects paid money to avoid forthcoming electric shocks of three different intensities, low, medium and high. The researchers found that subjects consistently paid more money to avoid pains that were greater than others in their recent history. In two sets of experiments, one where low shocks were mixed with medium shocks and one where medium shocks were mixed with higher ones, it was found that subjects paid much more money to buy out of medium shocks in the first condition than the second. Essentially, their evaluation of the undesirability of a particular magnitude of pain was contingent, not on the absolute magnitude of pain they were attempting to avoid, but on the set of pain options that they were forced to choose between.

Formally, the subject is first offered the choice set with the lower and medium pain options (LM), followed by further exposure to the choice set MH with the medium and high options. Assuming, reasonably, that the subject always prefers the option that provides the lesser amount of pain, their evaluation of relative (un)desirability of the medium option after experience with the choice set LM will be $R(M) = 1$. Upon further experience with MH, the new desirability of M will be,

$$\begin{aligned} R(M) &= \frac{p(r|M, LM)p(M|LM)p(LM) + p(r|M, MH)p(M|MH)p(MH)}{p(M|LM)p(LM) + p(M|MH)p(MH)}, \\ &= \frac{1 \times 1 \times p + 0 \times 1 \times (1-p)}{1 \times p + 1 \times (1-p)}, \\ &= \mathbf{p}, \end{aligned}$$

which, being less than 1, implies that the (un)desirability of M reduces after exposure to a higher degree of pain. This observation, while almost trite on surface, has eluded the descriptive abilities of utility function approaches of measuring value, as described comprehensively in [?].

Compromise effect

In the compromise effect, given a set of choices, {X,Y}, a subject prefers option X. Introduction of a third option Z leads to the development of two different ways of evaluating the desirability of any of the three items, resulting in situations where X may be strongly preferred to Z along one axis of measurement and strongly dominated by Z along the other.

In standard descriptions of this effect, these different ways of evaluation are regarded as attributes, leading to a simple description of the problem in the framework of multi-attribute utility theory. For our current purpose, we achieve the same purpose notationally by considering XY and YX to be two different observation contexts representing possibilities that always co-occur, but are not always evaluated identically. At the time of the first observation, the only possible contexts are XY and YX. Introduction of the third option, however, results in the (recalled) feasibility of six different contexts, which we index in $\mathcal{C} = \{XY, YX, YZ, ZY, ZX, XZ\}$. We denote the prior desirability imputed to objects in each of the six contexts as

$p(r\|X, XY)$	$p(r\|X, YX)$	$p(r\|Y, YZ)$	$p(r\|Y, ZY)$	$p(r\|Z, ZX)$	$p(r\|Z, XZ)$
x_1	x_2	y_1	y_2	z_1	z_2
$p(r\|Y, XY)$	$p(r\|Y, YX)$	$p(r\|Z, YZ)$	$p(r\|Z, ZY)$	$p(r\|X, ZX)$	$p(r\|X, XZ)$
$1 - x_1$	$1 - x_2$	$1 - y_1$	$1 - y_2$	$1 - z_1$	$1 - z_2$

Since the contexts ij and ji are indistinguishable as observable choice sets, they occur with the same sample frequency. Thus, we can assume a posterior belief on six contexts, $\{p_1, p_1, p_2, p_2, p_3, p_3\}$. When only X and Y are observed, the relative desirability of X will then be $\frac{x_1 \times p_1 + x_2 \times p_1}{p_1 + p_1} = 0.5(x_1 + x_2)$. Identically, the relative desirability of Y within the choice set $\{Y, Z\}$ can be written as $0.5(y_1 + y_2)$, and that of Z within the choice set $\{Z, X\}$ as $0.5(z_1 + z_2)$. Since we know that $X \succ Y$, $x_1 + x_2 > 1$. Also, since we know that no option dominates any other option along both dimensions, we can also say that $0.5 \leq x_1 + x_2 \leq 1.5, 0.5 \leq y_1 + y_2 \leq 1.5, 0.5 \leq z_1 + z_2 \leq 1.5$.

Now, upon observing XYZ, the desirability computation for X yields,

$$
\begin{aligned}
R(X) &= \frac{\sum_c^{\mathcal{C}} p(r|X, c)p(X|c)p(c)}{\sum_c^{\mathcal{C}} p(X|c)p(c)}, \\
&= \frac{x_1 p_1 + x_2 p_1 + 0 + 0 + (1 - z_1)p_3 + (1 - z_2)p_3}{p_1 + p_1 + 0 + 0 + p_3 + p_3}, \\
&= 0.5 \frac{(x_1 + x_2)p_1 + (2 - z_1 - z_2)p_3}{p_1 + p_3}.
\end{aligned}
$$

A similar computation for $R(Y)$ yields,

$$
\begin{aligned}
R(Y) &= \frac{\sum_c^{\mathcal{C}} p(r|Y,c)p(Y|c)p(c)}{\sum_c^{\mathcal{C}} p(Y|c)p(c)}, \\
&= \frac{(1-x_1)p_1 + (1-x_2)p_1 + y_1p_2 + y_2p_2 + 0 + 0}{p_1 + p_1 + p_2 + p_2 + 0 + 0}, \\
&= 0.5\frac{(2-x_1-x_2)p_1 + (y_1+y_2)p_2}{p_1+p_2}.
\end{aligned}
$$

An analytic comparison between these two quantities in their most general form is intractable. Here, we focus on determining a minimal and reasonable set of assumptions that analytically yields $R(Y) > R(X)$ and hence a reversal of the subject's original preference. The simplifying additional assumption we make is that objects that are extremely different (X and Z here) will have very little prior history of pairwise comparison. Hence, we assume that $p_3 \ll p_1, p_2$. For analytical simplicity, assume that $p_3 \approx 0$. Then, $2 \times R(X) \approx x_1 + x_2$, and $2 \times R(Y) \approx (2 - x_1 - x_2)p_1 + (y_1 + y_2)p_2$.

It is straightforward to see that $2 \times R(y) \leq \max(y_1 + y_2, 2 - x_1 - x_2)$. Since $2 \times R(x) = x_1 + x_2 > 1$, clearly $y_1 + y_2 > x_1 + x_2$ for any $R(Y) > R(X)$. For any set of objects that satisfy this criterion, there will exist some values of p_1 and $p_2 \approx 1 - p_1$ for which $R(Y) > R(X)$. In general, the greater the difference between the x sum and the y sum, the more probability points will satisfy our inequality. Numerical analysis across all possible values of $x_1 + x_2$ and $y_1 + y_2$ between 1 and 1.5 such that $y_1 + y_2 > x_1 + x_2$ tells us that, on average $p_1 < 0.39$ to ensure $R(Y) > R(X)$. We performed this analysis by sampling $R(X)$ and $R(Y)$ for $y_1 + y_2$ and $x_1 + x_2$ values at 10^{-4} intervals in the range $[1, 1.5]$ and finding the lowest value of p_1 for which $R(Y) > R(X)$. Computing the average of this probability for all points on our $10^{-4} \times 10^{-4}$ grid where $y_1 + y_2 > x_1 + x_2$ gives us 0.39 as the sample estimate.

Thus, if $y_1 + y_2 > x_1 + x_2$, $R(Y) > R(X)$ for a range of context probabilities. These two additional requirements for the compromise effect to emerge have simple interpretations: first, object Y should be more clearly preferable to object Z than object X is preferable to object Y - a cleanly testable prediction. Second, depending on how much more easily subjects will prefer Y to Z than X to Y, the secondary comparison between Y and Z will have to be more psychologically *accessible* in order to produce a preference reversal. These two assumptions may be seen as pieces of a single

fundamental requirement that the choice set YZ be substantially salient in a subject's mind, analogous to the $p > 1/2$ requirement in the similarity effect.
