# Supplementary material for: Learning What to Want: Context-Sensitive Preference Learning
Source: PLoS One. 2015 Oct 23;10(10):e0141129. doi: 10.1371/journal.pone.0141129 (PMC4619741; doi:10.1371/journal.pone.0141129)
Supplement: S4 File — (PDF) [file pone.0141129.s004.pdf]

The Allais paradox is still paradoxical

Here, we demonstrate the inability of our value inference theory to rationalize the famous Allais paradox (described in Table 1), suggesting that it does not yet capture the full richness of human preference dynamics.

1A	1B	2A	2B
$1000, 100% chance	$1000, 89%<br>$0, 1%<br>$5000, 10%	$0, 89%<br>$1000, 11%	$0, 90%<br><br>$5000, 10%

Table 1. The Allais paradox. Subjects that prefer option 1A to option 1B must rationally prefer option 2A to option 2B. However, empirical data shows that human subjects tend to prefer option 1A to 1B and option 2B to 2A in violation of normative expected utility maximization.

The Allais paradox maps on to our theory through the assumption that binary comparisons between the three quantities {0, 1000, 5000} yield observations that predict past comparative contexts. For instance, in contemplating the choice between options 2A and 2B, the comparative contexts {0, 0}, {1, 0} and {1, 5} are activated in the subject's inductive recollection, while the choice between 1A and 1B activates contexts {1, 1}, {1, 0} and {1, 5}. Along the lines of our earlier demonstrations, we can compute relative desirabilities as follows,

$$\begin{aligned}
R(2A) &= \frac{p(r|2A,\{0,0\})p(2A|\{0,0\})p(\{0,0\}) + \cdots}{p(2A|\{0,0\})p(\{0,0\}) + p(2A|\{1,0\})p(\{1,0\}) + p(2A|\{1,5\})p(\{1,5\})} \\
&= \frac{0.5 \times 1 \times 0.89 \times 0.90 + 1 \times 1 \times 0.11 \times 0.90 + 0 \times 1 \times 0.89 \times 0.1}{1 \times 0.89 \times 0.90 + 1 \times 0.11 \times 0.90 + 1 \times 0.89 \times 0.1}, \\
&= \mathbf{0.4995},
\end{aligned}$$

and,

$$\begin{aligned}
R(2B) &= \frac{p(r|2B,\{0,0\})p(2B|\{0,0\})p(\{0,0\}) + \cdots}{p(2B|\{0,0\})p(\{0,0\}) + p(2B|\{1,0\})p(\{1,0\}) + p(2B|\{1,5\})p(\{1,5\})} \\
&= \frac{0.5 \times 1 \times 0.89 \times 0.90 + 1 \times 1 \times 0.11 \times 0.90 + 0 \times 1 \times 0.89 \times 0.1}{1 \times 0.89 \times 0.90 + 1 \times 0.11 \times 0.90 + 1 \times 0.89 \times 0.1}, \\
&= \mathbf{0.5005},
\end{aligned}$$

which indicates that preferring option 2B is rational.

Performing a similar computation for the first gamble yields,

$$\begin{aligned} R(1A) &= 0.5 \times 1 \times 0.89 + 1 \times 1 \times \widehat{\mathbf{0.01}} + 0 \times 1 \times 0.1, \\ &= \mathbf{0.455}, \end{aligned}$$

and,

$$\begin{aligned} R(1B) &= 0.5 \times 1 \times 0.89 + 0 \times 1 \times \widehat{\mathbf{0.01}} + 1 \times 1 \times 0.1, \\ &= \mathbf{0.545}, \end{aligned}$$

yielding the conclusion that preferring option 1B is preferable, along classical utility maximization expectations, but *contra* the observed behavior of Allais paradox subjects. Again, resorting to prospect theory, it is easy to see that an over-estimate of greater than 10 for the 1% chance of obtaining nothing (highlighted above) in gamble 1B would harmonize the relative desirability calculation with the Allais paradox's predictions. Such an apparently drastic over-estimate of 1% empirical risk is empirically documented in human subject behavior. Thus, combining inferred desirability with the prospect theory probability weighting function potentially explains the origin of the Allais paradox, in the same way as combining EU with non-linear probabilities does in prospect theory.
